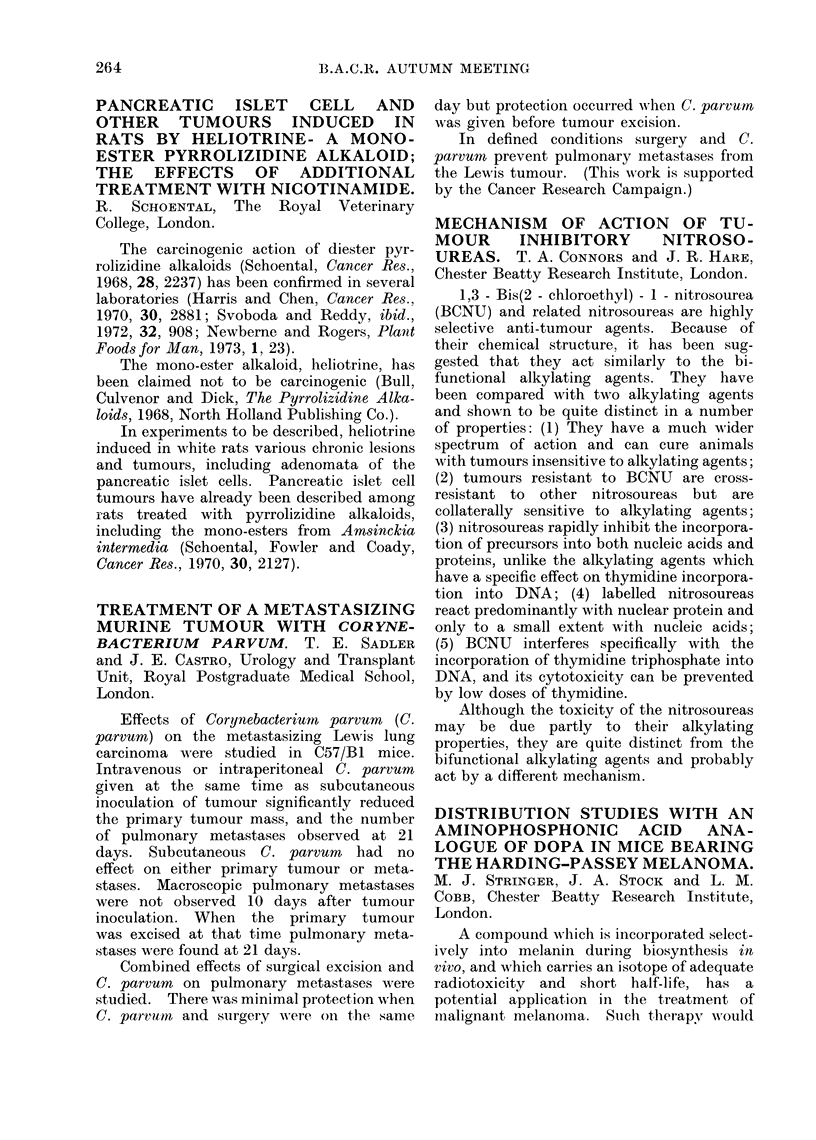# Proceedings: Pancreatic islet cell and other tumours induced in rats by heliotrine- a mono-ester pyrrolizidine alkaloid; the effects of additional treatment with nicotinamide.

**DOI:** 10.1038/bjc.1975.49

**Published:** 1975-02

**Authors:** R. Schoental


**PANCREATIC ISLET CELL AND OTHER TUMOURS INDUCED IN RATS BY HELIOTRINE- A MONO-ESTER PYRROLIZIDINE ALKALOID; THE EFFECTS OF ADDITIONAL TREATMENT WITH NICOTINAMIDE.** R. SCHOENTAL, The Royal Veterinary College, London.

The carcinogenic action of diester pyrrolizidine alkaloids (Schoental, *Cancer Res.*, 1968, **28**, 2237) has been confirmed in several laboratories (Harris and Chen, *Cancer Res.*, 1970, **30**, 2881; Svoboda and Reddy, *ibid.*, 1972, **32**, 908; Newberne and Rogers, *Plant Foods for Man*, 1973, **1**, 23).

The mono-ester alkaloid, heliotrine, has been claimed not to be carcinogenic (Bull, Culvenor and Dick, *The Pyrrolizidine Alkaloids*, 1968, North Holland Publishing Co.).

In experiments to be described, heliotrine induced in white rats various chronic lesions and tumours, including adenomata of the pancreatic islet cells. Pancreatic islet cell tumours have already been described among rats treated with pyrrolizidine alkaloids, including the mono-esters from *Amsinckia intermedia* (Schoental, Fowler and Coady, *Cancer Res.*, 1970, **30**, 2127).